# Review of "Biomedical Informatics; Computer Applications in Health Care and Biomedicine" by Edward H. Shortliffe and James J. Cimino

**DOI:** 10.1186/1475-925X-5-61

**Published:** 2006-11-21

**Authors:** Gari D Clifford

**Affiliations:** 1 M.I.T., 77 Massachusetts Av., Cambridge MA 02139, USA

## Abstract

This article is an invited review of the third edition of "Biomedical Informatics; Computer Applications in Health Care and Biomedicine", one of thirty-six volumes in Springer's 'Health Informatics Series', edited by E. Shortliffe and J. Cimino. This book spans most of the current methods and issues in health informatics, ranging through subjects as varied as data acquisition and storage, standards, natural language processing, imaging, electronic health records, decision support, teaching methods and ethics. The book is aimed at 'healthcare professionals', and is certainly appropriate for the non-technical informatics user. However, this book is also excellent background reading for the technical engineer who may be interested in the possible problems that confront the users in this field.

The impressive third edition of this enormous volume of twenty-four chapters and over a thousand pages is one of thirty-six volumes in Springer's 'Health Informatics Series' and ambitiously aims to span all of the current methods in health informatics. To do so, Professors Shortliffe and Cimino have assembled an excellent set of authors who themselves are among the major contributors to knowledge in this field. The series claims to be aimed at 'healthcare professionals', and this book is certainly appropriate for the non-technical informatics user. However, this book is also excellent background reading for the technical engineer who may be interested in the possible problems that confront the users in this field. In particular, this third edition provides many practical examples of the social and clinical context in which biomedical information technology is developed and implemented.

The book is divided into three units. Unit one is entitled 'recurrent themes in biomedical informatics', and consists of eleven chapters on subjects as varied as data acquisition and storage, standards, natural language processing, imaging, and ethics. Unit two also comprises eleven chapters and is devoted to biomedical informatics applications such as electronic health records, tele-health, patient monitors, imaging systems, decision support and teaching methods. Unit three is entitled 'biomedical informatics in the years ahead' and consists of two chapters, one on health care financing for information technology, and one on the future of computer applications in biomedicine. Each chapter is written in a 'continuing education' style, beginning with a list of bullet points listing the questions that you should be able to answer after reading the chapter. Most chapters are liberally scattered with pertinent diagrams, photographs, tables and practical examples to illustrate the points in the test (although some of the photographs of researchers 'in action' do not always add very much to the text). In general, the text is well laid out, clear and pertinent. The chapters then end with a list of further readings and discussion topics. The comprehensive list of references is given at the end of the book.

Chapter one by Shortliffe and Blois provides an interesting background and brief history of the discipline. In chapter two, Shortliffe and Barnett extend this history and rationale for the medical record. Chapter three is a rather excellent introduction to probabilistic clinical reasoning. In this chapter Owens and Sox explain simply and clearly the concepts of uncertainty, probability, sensitivity, specificity and clinical decision making in this context. My only disappointment with this chapter was that the authors did not provide an overview of the standard statistical techniques that are sometimes applied at will in the medical literature, without testing the underlying assumptions of the tests themselves (such as the form of the distributions). However, this subject could form a book in itself and indeed, the further reading suggests exactly this. (Such material is covered in chapter twenty-four in [1].) Chapter four by Patel and Kaufman, presents a high level overview of bioinformatic cognitive science, and chapter five, by Wiederhold and Rindfleisch, describes the relevant basics of computer-based data storage, access and transmission. In chapter six, Wiederhold and Shortliffe detail the importance and methods of human-computer interaction in the clinical context. Chapter seven, by Hammond and Cimino, addresses the extremely important topic of standards in biomedical informatics and although it provides an excellent overview of many of the standards and issues surrounding data storage, interchange and security, the chapter does gloss over one small and significant topic; the representation of high resolution biomedical signals such as the ECG and EEG. References are made to XML, (an excellent choice for annotation, which is not discussed), but no mention of its drawbacks in this context are given. There are numerous well-designed compact data formats that can deal with asynchronicity, missing data, security and transmission in an elegant and open manner. These issues are non-trivial and are often glossed over by a clinical audience. I would have been extremely interested to see a comparative analysis between biomedical signal and image standards, and a discussion of why DICOM was so readily accepted in the imaging field, yet despite obvious contenders, no single parallel standard has evolved in biomedical signals as yet. Friedman and Johnson present an excellent overview of natural language and text processing in chapter eight and Brinkley and Greenes provide a similar overview of imaging techniques in chapter nine. In chapter ten, Goodman and Miller address the important issue of ethics and legality in health informatics. Friedman, Wyatt and Owens tackle the tricky issue of evaluation and performance of health care technology in chapter eleven.

In chapter twelve, the first chapter in unit two, Tang and McDonald describe the concept of an electronic medical record and provide numerous examples, describing the concept's rationality and utility. In chapter thirteen Vogel, Safran and Perreault present an overview of the management of information in healthcare organizations, with an emphasis on historical developments over time. In chapter fourteen, Brennan and Starren address the motivation and requirements for successful consumer health care and telemedicine. Although much of the book is devoted to the organization of health care systems, it is refreshing to see a specific chapter (fifteen by Yasnoff, O'Carroll and Friede) on public health information systems. Chapter sixteen by Ozbolt and Bakken draws a distinction between patient care systems and the patient data, but sometimes presents information that can be found in earlier chapters. This is somewhat inevitable in book with so many authors and so many common themes, and cross-referencing of other chapters is frequent. In chapter seventeen, Gardner and Shabot present an excellent overview of patient monitoring systems with a focus on data integration, a chapter anyone dealing with data in the ICU should read. Chapter eighteen by Greenes and Brinkley provides a wide overview of the issues surrounding the use of radiology imaging systems. In chapter nineteen Hersh, Stavri and Detmer detail the variety of online medical databases available and methods for searching them. However, many of the significant online medical information databases are not mentioned. In chapter twenty, Musen, Shahar and Shortliffe detail examples of clinical decision-support systems, and a system for designing them. Chapter twenty-one, Dev, Hoffer and Barnett address the issues surrounding the use of computers in medical education. The final chapter in unit two, by Altman and Mooney, presents an overview of genetic informatics. Chapter twenty-three by Singer, Enthoven and Garber, provides a historical perspective of health care financing and information technology. Fagan and Shortliffe conclude the book with a chapter on the future of information technology in biomedicine by discussing current trends in technology, financing, legal issues and medical culture.

One important point (among many) that is made in the preface is that there is a growing need for specialists in biomedical informatics who can bridge the gap between clinical practice and the creators of the technology. There are, of course, two routes to this; clinicians who are willing to learn more about the technology, and technologists who are willing to learn more about the environment in which the technology is employed. This book provides an isthmus between these worlds.

Although the minimal use of equations in this text makes this book mainly appropriate for the clinical practitioner, it is also excellent reading for anyone interested in the field, its development and the outstanding challenges. For a field that is constantly in flux at a rate tied to the complex dynamic between the rate of development of technology, the profit of the companies involved and the politics of regulation, the authors and editors are to be congratulated on continuing to provide up-to-date sign posts and maps for our biomedical orienteering.

Further reading can be found in [1-5] and excerpts from the book can be found at [6].
